# Music Therapy in Pain and Anxiety Management during Labor: A Systematic Review and Meta-Analysis

**DOI:** 10.3390/medicina56100526

**Published:** 2020-10-10

**Authors:** Rocio Santiváñez-Acosta, Elena de las Nieves Tapia-López, Marilina Santero

**Affiliations:** 1 Centro Nacional de Salud Intercultural, Instituto Nacional de Salud, Av. Defensores del Morro 2268 (Ex Huaylas)—Chorrillos, Lima 9, Lima 15066, Peru; 2 Universidad Peruana Cayetano Heredia, Av. Honorio Delgado 430, San Martín de Porres, Lima 15102, Peru; elena.tapia.l@upch.pe; 3 Instituto de Efectividad Clínica y Sanitaria (IECS), Escuela de Salud Pública de la Universidad de Buenos Aires, Marcelo T. de Alvear, Buenos Aires 2202, Argentina; msantero@iecs.org.ar

**Keywords:** systematic review, meta-analysis, labor pain, anxiety, music therapy, mind–body therapies (source: MeSH)

## Abstract

*Background and Objective:* The study of music therapy in labor is unknown. The main objective of this research was to evaluate the effectiveness of music therapy to manage pain and anxiety during labor. *Materials and Methods:* A search strategy was used with PubMed/MEDLINE, LILACS, Cochrane, TRIPDATABASE, and Google Scholar. The selection criteria were based on randomized clinical trials; quasi-experimental research on pain intensity and anxiety during labor was evaluated. The primary outcomes were measured by the Visual Analogue Scale (VAS). A meta-analysis of the fixed effects was performed using mean differences (MD). Twelve studies were included for the final analysis, six (778 women) of which were meta-analyzed. *Results:* Decreased VAS scores for pain intensity associated with music therapy were found in the latent (MD: −0.73; 95% CI −0.99; −0.48) and active (MD: −0.68; 95% CI −0.92; –0.44) phases of labor. VAS scores for anxiety decreased both in the latent (MD: −0.74; 95% CI −1.00; −0.48) and active (MD: −0.76; 95% CI −0.88; −0.64) phases. *Conclusion:* Music therapy seems to have beneficial effects on pain intensity and anxiety during labor, especially for women giving birth for the first time. However, the evidence is qualified as low.

## 1. Introduction

The World Federation of Music Therapy defines music therapy as the use of music and/or musical elements (sound, rhythm, melodies, or harmonies) to ease and promote communication, relationships, learning, movement, expression, organization, and other relevant therapeutic objectives, thereby solving physical, emotional, mental, social, and cognitive needs [1]. Both medicine and music are capable of being used in order to improve the human condition and their union gives rise to so-called music therapy—that is, therapy through music [2]. It is currently used as a complementary therapy for physical, mental, and surgical procedures [3]. Music therapy seeks to develop, in a culturally accepted way, the potential and/or abilities of patients, in an effort to ensure better intrapersonal/interpersonal integration and subsequent improved quality of life through prevention, rehabilitation, and treatment [4].

The effects of music therapy have been studied for the gestation and delivery periods. Studies have shown promising results of decreasing levels of anxiety and stress in the mother [5,6], and improved fetal parameters (such as heart beat variability) [7]. However, these results cannot be extrapolated to the general population due to the degree of variability between the studies, low number of patients evaluated, and the risk of bias [8].

Peru recognizes music therapy as a part of its services provided during obstetrical psycho-prophylaxis, especially during sessions that instruct the mother on how to prepare for childbirth and the postpartum period [9], and also as mind–body therapy is included in the health system [10]. In pursuit of comprehensive and timely care during childbirth, this systematic review and meta-analysis seeks to evaluate the effectiveness of music therapy in the management of pain and anxiety during labor.

This study sought to answer the following research questions: (a) How effective is music therapy in pain and anxiety management during labor? (b) How effective is music therapy in reducing pain and anxiety, when compared to standard care? (c) How effective is music therapy in alleviating pain, as measured by lowered vital signs—i.e., heart rate, respiration rate, and systolic and diastolic blood pressure), when compared to standard care? (d) Is there a difference in the effectiveness of music therapy in reducing pain and anxiety in primiparous women during the active and latent phases, when compared to standard care?

## 2. Materials and Methods

Previous protocol was not registered or published for this systematic review and meta-analysis. We used the PICOT framework (Patients, Intervention, Comparison, Outcomes, Type of study) to guide our eligibility criteria. This systematic review and meta-analysis included randomized clinical trials and/or quasi-experimental studies with complete and accessible information. These studies evaluated the effect of music therapy in the final months of pregnancy on labor, compared to the control groups (without music therapy or offered other types of therapies). Observational studies, case reports, and book chapters were excluded. We considered studies included women with no health problems, and no discrepancy in gestation number, age, type of pregnancy (single or multiple), or delivery method (vaginal or Caesarean section). The primary outcomes evaluated were pain intensity and anxiety, measured by Visual Analog Scale (VAS). Additionally, other types of outcomes were considered; in the case of the mother, satisfaction, depression, systolic or diastolic blood pressure, medication use, maternal heart rate; in the case of the newborn, Apgar, heart rate, admission to the intensive care unit (ICU). Finally, information on safety and/or adverse events was also collected. Only studies in English or Spanish were included.

The systematic search of the scientific literature was carried out using bibliographic databases such as PubMed/Medline, LILACS, and COCHRANE, and search engines such as TRIPDATABASE and Google Scholar. The use of keywords, descriptors, and Boolean operators was sought, covering everything published from 2003 until June 2018—84 studies were thus obtained. An additional 11 studies were identified from secondary references and gray literature. On eliminating duplicates, 91 studies were finally selected.

The studies were selected by two reviewers, part of the research team (E.N.T.-L. and M.S.), who independently evaluated the studies according to inclusion criteria and organized them by title and summary using EndNote X7.2.1^®^ Reference Manager. Any doubts or disagreements were resolved between the reviewers, and a third opinion was requested by the team investigator (R.S.-A.) in the case of a disagreement.

The data were extracted independently by the reviewers, followed by a second review by the research team in order to improve and guarantee the quality of the process. Subsequently, the risk of bias was assessed using a quality tool validated by The Cochrane Collaboration^®^ [11]. The studies were rated as low risk, high risk, or uncertain risk of bias in six domains: random sequence generation (selection bias), allocation concealment (selection bias), blinding of participants and staff (detection bias), blinding of outcome assessors (detection bias), incomplete outcome data (attrition bias), and selective reporting of results (reporting bias).

With the help of the Review Manager (RevMan), version 5.3, and the Cochrane Collaboration^®^, we figured a meta-analysis of fixed effects for the main outcomes, obtaining mean differences (MD) with 95% confidence intervals (95% CI); an effect with a *p* ˂ 0.05 value was considered statistically significant. The I2 statistic and chi-square tests were used to measure heterogeneity. Additionally, summary tables were used to interpret the findings, using the GRADE criteria to classify the quality of the evidence found (high, moderate, low, or very low).

Finally, narrative evaluations of the findings were carried out in subgroups—number of pregnancies, route of birth, type of music used, phase of delivery, and postpartum stage (puerperium).

## 3. Results

**Figure 1:**
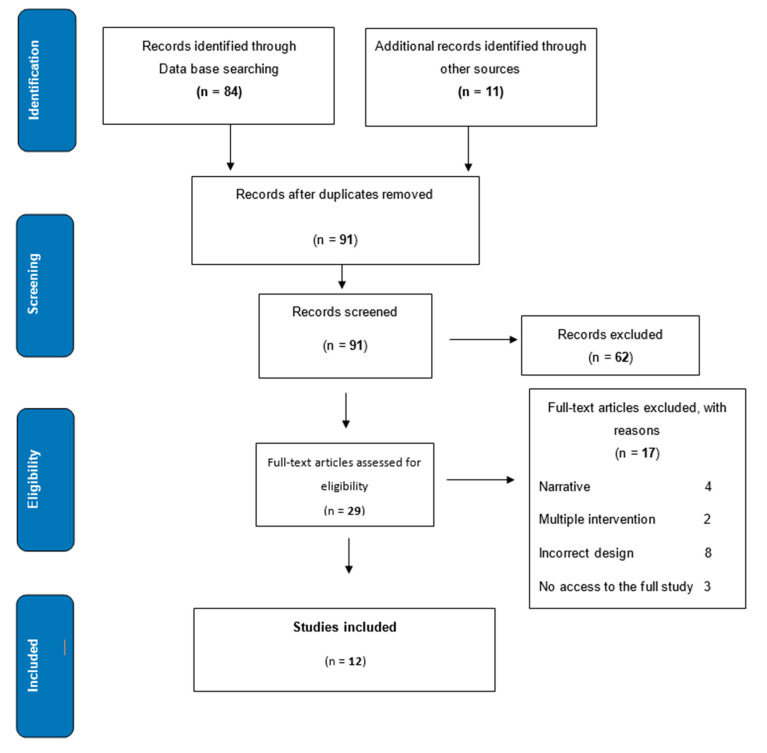
PRISMA flow chart.

Between 2003 and June 2018, 91 studies were identified using a search strategy. Of these, 62 were excluded, and 12 studied that met all the criteria (all in English) were finally chosen (Figure 1) for this research work. More than half (9/12, 58.3%) of the evaluated patients had had a normal delivery. In some studies, the control group was offered another form of intervention; for example, massages (33.3%). However, the majority of studies (8/12, 66.7%) used standard therapy or care as a control, as per the place where the study was conducted (Table 1).

**Figure 2:**
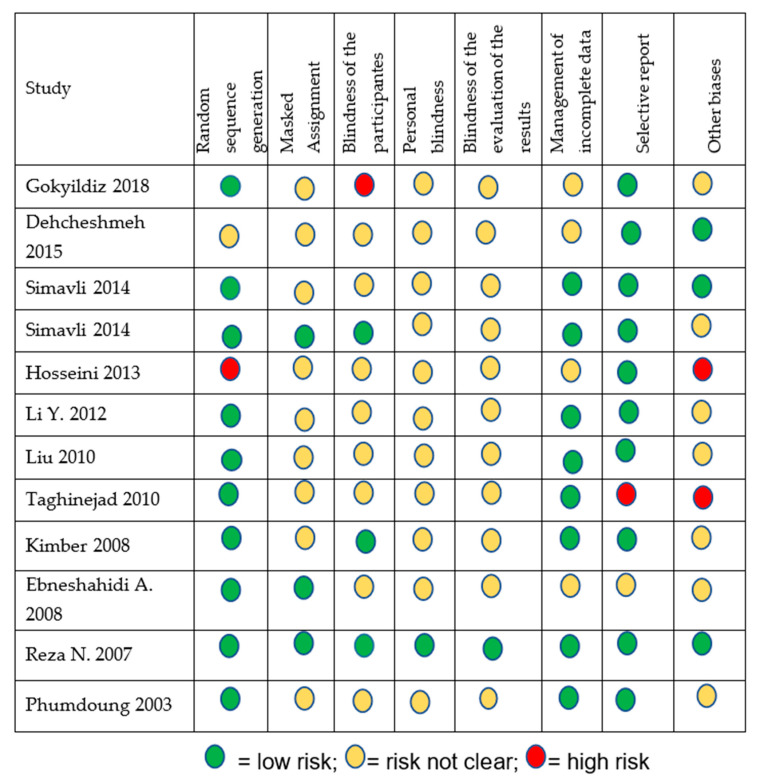
Risk of bias; review of the authors’ judgments on each element.

Most of the studies were found to be of moderate to low quality, with an uncertain level of bias, as the masking and randomization procedure was generally not described (Figure 2).

### 3.1. Effect of Music Therapy on Labor Pain

**Figure 3:**
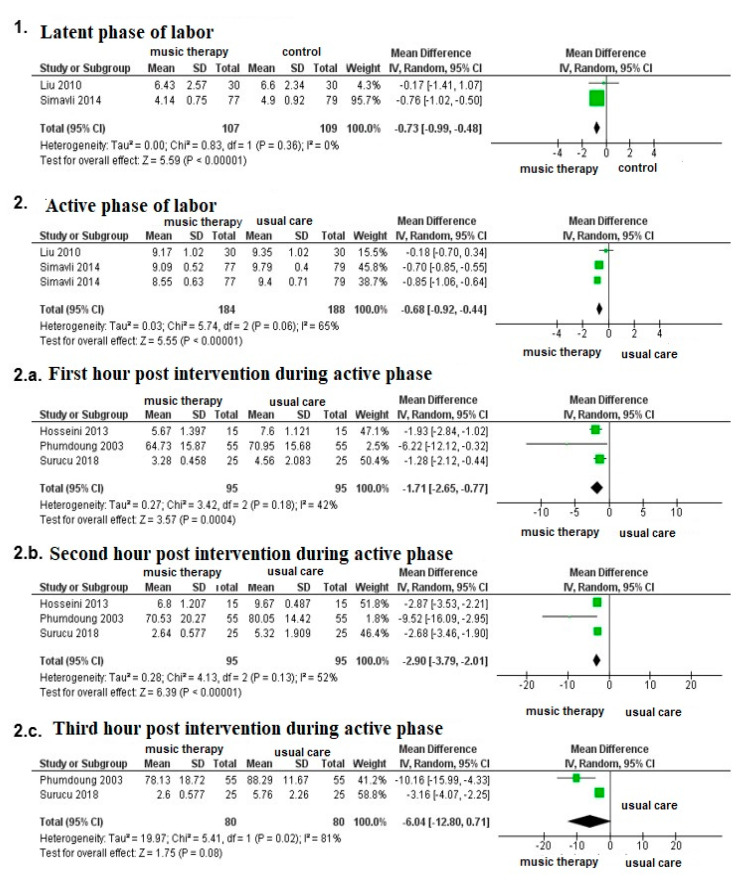
Meta-analysis of the effect of music therapy on the intensity of pain during the latent and active phases of labor.

With respect to studies that only evaluated primiparous women, the results highlight the benefits of music therapy, as compared to standard therapy, although the intervention should be measured heterogeneously. Despite this, the meta-analysis showed significant differences in VAS scores, favoring music therapy in the intensity of latent pain (MD: −0.73; 95% CI −0.99; −0.48); in the active phase (MD: −0.68; 95% CI −0.92; −0.44) in its entirety or during the first phase (MD: −1.71; 95% CI −2.65; −0.77) and second hour postintervention (MD: −2.90; 95% CI −3.79; −2.01) (Figure 3).

Only two studies evaluated the use of music therapy in the immediate postpartum period (14, 15). These studies showed statistically significant results from immediate to 24 h postpartum; however, the heterogeneity and presence of bias made it impossible to perform a meta-analysis. On the other hand, only one study evaluated changes in pain intensity with music therapy [20], and no significant differences were found during labor; however, the specific time of labor, how the intervention was performed, or how long it was evaluated for was not specified.

Only three studies evaluated the effect of music therapy on the intensity of post-Cesarean pain [17,21,22], finding significant decreases in VAS—between 1.6 and 1.9. However, heterogeneity in investigational methods prevented a meta-analysis from being conducted.

### 3.2. Effect of Music Therapy on Anxiety during Labor

Of the twelve studies included in this systematic review (RS), eight evaluated anxiety levels (66.7%). All the studies that evaluated the effect of music therapy on the level of anxiety had regular care as control.

**Figure 4:**
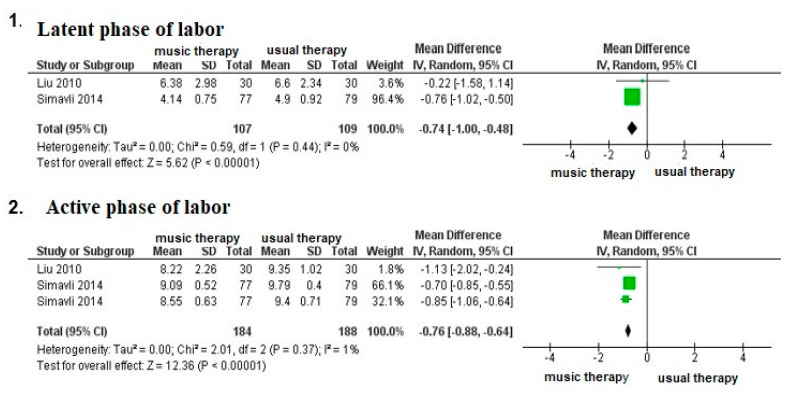
Meta-analysis on the effect of music therapy on anxiety during the latent and active phases of labor.

In primiparous patients in labor, four studies evaluated the outcome of anxiety; a meta-analysis was conducted with three of them (14,15,18). The results revealed significant differences in the use of music therapy in the latent phase (MD: −0.74; 95% CI −1.00; −0.48) and in the active phase of labor (MD: −0.76; 95% CI −0.88; −0.64) (Figure 4). Likewise, similar to the pain results, one study evaluated the differences in anxiety levels measured with a VAS in the postpartum period in primiparous patients, finding significant differences in the benefits of music therapy one hour to 24 h postpartum [12].

In three studies, differences in anxiety after a Cesarean section as a result of music therapy in the preoperative period were evaluated [17,21,22]. A study by Li et al. [15] found a decrease in anxiety symptoms up to 6 h in the postoperative period; however, this variable was measured with the self-reported anxiety scale (SAS score). On the other hand, studies by Ebneshahidi et al. [21] and de Reza et al. [22] found no significant differences in assessing anxious symptoms using VAS.

### 3.3. Quality of Evidence Found

The evidence gathered in respect to the effect of music therapy on labor pain was found to be of low quality, except for the subgroup of patients evaluated during the active phase, where the evidence in favor of music therapy was rated as moderate.

Similarly, for the outcome that evaluated the effect of this intervention on anxiety during labor, the quality of the evidence was rated as moderate when the studies investigated the effect of music therapy during the active phase (Table 2).

### 3.4. Other Secondary Outcomes

Only one study addressed the effect of music therapy on the scores of the Edinburgh Postpartum Depression Scale (EPDS), finding significant differences in favor of this therapy in assessments on the first and eighth days of the puerperium period [14].

On the other hand, four studies reported results on medication consumption: Kimber et al. [14] reported a 10% decrease in the use of medicine, compared to those who received standard therapy [20]; however, this decline was not statistically significant. In the remaining three studies, a specific group of analgesics was addressed—Ebneshahidi et al. [21] found that morphine use 30 min after a Cesarean section was significantly lower in the group that received music therapy (*p* ˂ 0.05) [22]. In addition, Simavli et al. [15] showed a lower consumption of paracetamol and diclofenac in the postoperative period (between 8–24 h) in patients receiving music therapy (15).

In the case of maternal cardiovascular parameters (blood pressure and heart rate), the studies had conflicting results. For example, Simavli et al. [15] showed promising results in respect to a decrease in maternal heart rate and systolic blood pressure in the active phase and up to two hours postpartum, compared to those receiving usual care [15]. The studies by Gokyildiz et al. [12] and Ebneshahidi et al. [21] also showed favorable results, but were not statistically significant [12,21].

Finally, only the study by Gokyildiz et al., 2018, evaluated the evolution of fetal heart rate in women who had received music therapy in the active phase (1–7 h), without finding significant differences [12].

### 3.5. Safety and Adverse Effects of Music Therapy

None of the 12 studies reported any adverse effects or unfavorable outcomes with music therapy during labor.

## 4. Discussion

This review highlights the benefits of music therapy in the management of pain and anxiety levels, especially in primiparous women, during the latent and active phases of labor. However, the heterogeneity and degrees of bias prevent us from detecting significant and clinically important differences (as compared to the control) that allow a clear recommendation of this practice.

In the case of variation in labor pain intensity, the results are consistent with those reported by Smith et al. [24], who addressed pain management by comparing music therapy and therapeutic massages [24]. On the other hand, Smith et al. [8], in a more recent systematic review, found discrete effects of music therapy on pain intensity, finding a difference in means (MD) of −0.73 (95% CI −1.01; −0.45) by conducting a meta-analysis of two studies (192 women) [8]. In comparison with the systematic review, this paper addresses three studies (372 women) and evaluates the effects by phases of labor, finding an MD of −0.73 (95% CI −0.99; −0.48) in the latent phase and −0.68 (95% CI −0.92; −0.44) in the active phase.

Regarding anxiety management, we did not find systematic reviews that evaluated the effect of music therapy on this variable. However, it should be noted that this is a fairly well-reported study variable in previous studies and in those published after the search period established for this paper. For instance, Garcia et al. [5], in a recent study, reports statistically significant decreases in the anxiety levels of pregnant women who performed a non-stressful test (NST) [5] in the third trimester. However, Teckenberg et al. [7], when evaluating the effect of this intervention on hospitalized pregnant women, found significant results only in patients who had high levels of anxiety prior to the intervention [7].

The secondary outcomes also showed contradictory results; however, the quality of the evidence found and heterogeneity in data collection did not allow for the realization of a meta-analysis or the elaboration of conclusive conclusions about the effects of music therapy on these outcomes.

It is important to note that the analysis by subgroups, despite not having achieved an increase in the possibilities of meta-analysis, offers characteristics not found in other studies. The study of intervening factors, such as labor phase, number of previous deliveries, and delivery method, allows the obtained results to be applied (with corresponding limitations) to a real clinical context in health facilities that offer these services. Therefore, it is recommended that future revisions consider these analyses in their designs and expand them according to the need observed in their populations.

Another limitation of this study is that within its objectives, it was not possible to evaluate the true effect of music therapy on the duration of labor phases; thus, future studies should consider collecting information on this variable.

The quality of the evidence found was the greatest limitation in obtaining conclusions that could fully answer the research question, as the GRADE evaluation rated the evidence as being of low to moderate quality, mainly due to heterogeneity levels and the high risk of bias found. Therefore, it is recommended that more randomized clinical trials with better quality are carried out, and that comply with reporting bias control systems (or, if unavoidable, all possible biases should enable a more real rating of the quality of evidence being reported).

Finally, no adverse effects or unfavorable outcomes of the complementary therapy were found, implying that it can be recommended to attending physicians as an addition to routine treatment, as long as health establishments have the required environments and professionals to provide the service.

## 5. Conclusions

This paper concludes that music therapy could have beneficial effects on the management of pain and anxiety during labor, preferably for primiparous women. However, the risk of inclusion of biases and limited number of trials prevents a strong recommendation from being made.

## Authors Contributions

R.S.-A. participated in the conception, study design, critical review of the manuscript and performed the data analysis. E.d.l.N.T.-L. and M.S. prepared the databases for the analysis and drafted the first version of the manuscript, in addition to performing the data analysis. All authors have read and agreed to the published version of the manuscript.

## Figures and Tables

**Table 1:** General characteristics of the studies included in this systematic review.

Study	*n*	Comparator	Patients	Type of Delivery	Kind of Music	Evaluation
Gokyildiz (2018) [12]	50	Conventional therapy	Primiparous	Normal delivery	Acemasiran	Labor (active phase) and postpartum
Dehcheshmeh (2015) [13]	90	Hoku point ice massage and conventional therapy	Primiparous	Normal delivery	Sounds of the sea	Labor (active phase)
Simavli (2014) [14]	156	Conventional therapy	Primiparous	Both of them	Classical, Turkish artistic music, Turkish folklore, Turkish classical and popular music	Labor (latent phase and active phase) and postpartum
Simavli (2014) [15]	161	Conventional therapy	Primiparous	Normal delivery	Classical, light, popular, artistic Turkish or Turkish folk music and Turkish Sufi music.	Postpartum
Hosseini (2013) [16]	30	Conventional therapy	Primiparous	Normal delivery	Relaxing music	Labor (active phase)
Li (2012) [17]	60	Conventional therapy	Not specified	Cesarean delivery	Not specified	Post caesarean
Liu (2010) [18]	103	Conventional therapy	Primiparous	Normal delivery	Five types of relaxing music	Labor (latent phase and active phase)
Taghinejad (2010) [19]	101	Massage	Primiparous	Normal delivery	Classic	Labor (active phase)
Kimber (2008) [20]	90	Massage	Both of them	Both of them	Not specified	Labor (active phase)
Ebneshahidi (2008) [21]	77	Conventional therapy	Not specified	Cesarean delivery	Not specified	Post Caesarean
Reza (2007) [22]	100	White music	Not specified	Cesarean delivery	Spanish guitar	Post Caesarean
Phumdoung (2003) [23]	110	Conventional therapy	Primiparous	Normal delivery	Five types of soft music	Labor (active phase)

**Table 2:** Summary of evidence on the effect of music therapy on pain and anxiety management during labor.

Outcomes	Absolute Anticipated Effects * (95% CI)		Relative Effect (95% CI)	No. of Participants (Studies)	Certainty of the Evidence (GRADE)
	Risk with or without Therapies	Risk with Music Intervention for Pain and Anxiety Management			
Labor Pain					
Latent labor pain		The average pain during latent labor in the intervention group was 0.73 lower (0.99 less than 0.48 less)	-	2 Randomized controlled experiments (RCTs)	⨁◯◯◯ VERY LOW
Active labor pain		The mean pain in the active phase of labor in the intervention group was 0.68 lower (0.92 less than 0.44 less)	-	3 RCTs	⨁⨁⨁◯ MODERATE
Pain in the first hour postintervention during the active phase of labor		The average pain in the first hour after intervention during the active phase of labor in the intervention group was 1.71 lower (2.65 less than 0.77 less)	-	3 RCTs	⨁⨁◯◯ LOW
Pain in the second hour postintervention during the active phase of labor		The average pain in the second hour postintervention during the active phase of labor in the intervention group was 2.9 lower (3.79 less than 2.01 less)	-	3 RCTs	⨁⨁◯◯ LOW
Pain in the third hour postintervention during the active phase of labor		The average pain in the third hour postintervention during the active phase of labor in the intervention group was 6.04 lower (12.8 lower than 0.71 higher)	-	2 RCTs	⨁⨁◯◯ LOW
**Anxiety during Labor**					
Latent labor anxiety		The average latent phase anxiety of labor in the intervention group was 0.74 lower (1 lower than 0.48 lower)	-	2 RCTs	⨁◯◯◯ VERY LOW
Anxiety in active phase of labor		The average anxiety during labor phase in the intervention group was 0.76 lower (0.88 lower than 0.64 lower)	-	3 RCTs	⨁⨁⨁◯ MODERATE

* The risk in the intervention group (and its 95% confidence interval) is based on the risk assumed in the comparison group and the relative effect of the intervention (and its 95% confidence interval). CI: Confidence Interval; Grades of evidence from the GRADE Working Group; High: High confidence in the match between the actual and estimated effect; Moderate: Moderate confidence in the estimation of the effect. There is a possibility that the actual effect is far from the estimated effect; Low: Limited confidence in the estimation of the effect. The actual effect may be far from the estimate; Very low: Little confidence in the estimated effect. The true effect is most likely different from the estimate.

## References

[1] Vink A., Hanser S. (2018). Music-Based Therapeutic Interventions for People with Dementia: A Mini-Review. Medicina.

[2] Miranda Marcelo C., Hazard Sergio O., Miranda Pablo V. (2017). La música como una herramienta terapéutica en medicina. Rev. Chil. Neuro-Psiquiatr..

[3] Kamioka H., Tsutani K., Yamada M., Park H., Okuizumi H., Tsuruoka K., Honda T., Okada S., Park S.-J., Kitayuguchi J. (2014). Effectiveness of music therapy: A summary of systematic reviews based on randomized controlled trials of music interventions. Patient Prefer. Adherence.

[4] Benenzon R. (2000). Musicoterapia De la Teoría a la Práctica.

[5] González J.G., Miranda M.I.V., Mullor M.R., Carreño T.P., Rodriguez R.A. (2017). Effects of prenatal music stimulation on state/trait anxiety in full-term pregnancy and its influence on childbirth: A randomized controlled trial. J. Matern. Neonatal Med..

[6] Hepp P., Hagenbeck C., Gilles J., Wolf O.T., Goertz W., Janni W., Balan P., Fleisch M., Fehm T., Schaal N.K. (2018). Effects of music intervention during caesarean delivery on anxiety and stress of the mother a controlled, randomised study. BMC Pregnancy Childbirth.

[7] Teckenberg-Jansson P., Turunen S., Pölkki T., Lauri-Haikala M.-J., Lipsanen J., Henelius A., Aitokallio-Tallberg A., Pakarinen S., Leinikka M., Huotilainen M. (2019). Effects of live music therapy on heart rate variability and self-reported stress and anxiety among hospitalized pregnant women: A randomized controlled trial. Nord. J. Music. Ther..

[8] Smith C.A., Levett K.M., Collins C.T., Armour M., Dahlen H.G., Suganuma M. (2018). Relaxation techniques for pain management in labour. Cochrane Database Syst. Rev..

[9] Ministerio de Salud (2012). Guía Técnica para la Psicoprofilaxis Obstétrica y Estimulación Prenatal.

[10] Santiváñez R., Condori C., Loayza M., Vásquez P., Valeriano L., Instituto Nacional de Salud (2016). Manual de Registro y Codificación de Actividades en la Atención de Medicina Alternativa y Medicina Complementaria. Serie de Manuales HIS N°05.

[11] Cumpston M., Li T., Page M.J., Chandler J., Welch V.A., Higgins J.P., Thomas J. (2019). Updated guidance for trusted systematic reviews: A new edition of the Cochrane Handbook for Systematic Reviews of Interventions. Cochrane Database Syst. Rev..

[12] Gokyildiz Surucu S., Ozturk M., Avcibay Vurgec B., Alan S., Akbas M. (2018). The effect of music on pain and anxiety of women during labour on first time pregnancy: A study from Turkey. Complement. Ther. Clin. Pract..

[13] Dehcheshmeh F.S., Rafiei H. (2015). Complementary and alternative therapies to relieve labor pain: A comparative study between music therapy and Hoku point ice massage. Complement. Ther. Clin. Pract..

[14] Simavli S.A., Kaygusuz I., Gumus I., Usluogulları B., Yildirim M., Kafali H., Usluogullari B., Yildirim M. (2014). Effect of music therapy during vaginal delivery on postpartum pain relief and mental health. J. Affect. Disord..

[15] Simavli S.A., Gumus I., Kaygusuz I., Yildirim M., Usluogullari B., Kafali H. (2014). Effect of Music on Labor Pain Relief, Anxiety Level and Postpartum Analgesic Requirement: A Randomized Controlled Clinical Trial. Gynecol. Obstet. Investig..

[16] Hosseini S.E., Bagheri M., Honarparvaran N. (2013). Investigating the effect of music on labor pain and progress in the active stage of first labor. Eur. Rev. Med. Pharmacol. Sci..

[17] Li Y., Dong Y. (2012). Preoperative music intervention for patients undergoing cesarean delivery. Int. J. Gynecol. Obstet..

[18] Liu Y.-H., Chang M.-Y., Chen C.-H. (2010). Effects of music therapy on labour pain and anxiety in Taiwanese first-time mothers. J. Clin. Nurs..

[19] Taghinejad H., Delpisheh A., Suhrabi Z. (2010). Comparison between massage and music therapies to relieve the severity of labor pain. Womens Health.

[20] Kimber L., McNabb M., Court C., Haines A., Brocklehurst P. (2008). Massage or music for pain relief in labour: A pilot randomised placebo controlled trial. Eur. J. Pain.

[21] Ebneshahidi A., Mohseni M. (2008). The Effect of Patient-Selected Music on Early Postoperative Pain, Anxiety, and Hemodynamic Profile in Cesarean Section Surgery. J. Altern. Complement. Med..

[22] Reza N., Ali S.M., Saeed K., Abul-Qasim A., Reza T.H. (2007). The impact of music on postoperative pain and anxiety following cesarean section. Middle East J. Anaesthesiol..

[23] Phumdoung S., Good M. (2003). Music reduces sensation and distress of labor pain. Pain Manag. Nurs..

[24] Smith C.A., Levett K.M., Collins C.T., Dahlen H.G., Ee C.C., Suganuma M. (2018). Massage, reflexology and other manual methods for pain management in labour. Cochrane Database Syst. Rev..

